# Identification of Clinical Phenotypes and Related Survival in Patients with Large HCCs

**DOI:** 10.3390/cancers13040592

**Published:** 2021-02-03

**Authors:** Brian I. Carr, Vito Guerra, Rossella Donghia, Fabio Farinati, Edoardo G. Giannini, Luca Muratori, Gian Ludovico Rapaccini, Maria Di Marco, Eugenio Caturelli, Marco Zoli, Rodolfo Sacco, Ciro Celsa, Claudia Campani, Andrea Mega, Maria Guarino, Antonio Gasbarrini, Gianluca Svegliati-Baroni, Francesco Giuseppe Foschi, Elisabetta Biasini, Alberto Masotto, Gerardo Nardone, Giovanni Raimondo, Francesco Azzaroli, Gianpaolo Vidili, Maurizia Rossana Brunetto, Franco Trevisani

**Affiliations:** 1 Translational HCC Research Department, Liver Transplant Institute, Inonu University, Malatya 44000, Turkey; 2 Clinical Trials Department, National Institute of Digestive Diseases, IRCCS S. de Bellis Research Hospital, 70013 Castellana Grotte, Italy; vmbguerra@hotmail.it (V.G.); rossydonghia@gmail.com (R.D.); 3 Department of Surgery, Oncology and Gastroenterology, University of Padova, 35122 Padova, Italy; fabio.farinati@unipd.it; 4 Department of Internal Medicine, Gastroenterology Unit, University of Genova, IRCCS Ospedale Policlinico San Martino, 16132 Genova, Italy; egiannini@unige.it; 5 Internal Medicine–Piscaglia Unit, Azienda Ospedaliero-Universitaria S. Orsola-Malpighi, 40138 Bologna, Italy; luca.muratori4@studio.unibo.it; 6 Gastroenterology Unit, Fondazione Policlinico Universitario A. Gemelli, IRCCS, 00168 Roma, Italy; gianludovico.rapaccini@unicatt.it; 7 Medicine Unit, Bolognini Hospital, 24068 Seriate, Italy; marielladimarco@gmail.com; 8 Gastroenterology Unit, Belcolle Hospital, 01100 Viterbo, Italy; e.caturelli@tiscali.it; 9 Department of Medical and Surgical Sciences, Internal Medicine–Zoli Unit, Alma Mater Studiorum—University of Bologna, 40126 Bologna, Italy; marco.zoli@unibo.it; 10 Gastroenterology and Digestive Endoscopy Unit, Foggia University Hospital, 71122 Foggia, Italy; saccorodolfo@hotmail.com; 11 Department of Health Promotion, Mother & Child Care, Internal Medicine & Medical Specialties, PROMISE, Gastroenterology & Hepatology Unit, University of Palermo, 90133 Palermo, Italy; celsaciro@gmail.com; 12 Department of Experimental and Clinical Medicine, Internal Medicine and Hepatology Unit, University of Firenze, 50121 Firenze, Italy; claudiacampani.cc@gmail.com; 13 Gastroenterology Unit, Bolzano Regional Hospital, 39100 Bolzano, Italy; andrea.mega@sabes.it; 14 Department of Clinical Medicine and Surgery, Gastroenterology Unit, University of Napoli “Federico II”, 80138 Napoli, Italy; maria.guarino@unina.it; 15 Internal Medicine and Gastroenterology Unit, Policlinico Gemelli, Università Cattolica del Sacro Cuore, 00168 Roma, Italy; antonio.gasbarrini@unicatt.it; 16 Liver Injury and Transplant Unit, Polytechnic University of Marche, 60121 Ancona, Italy; g.svegliati@univpm.it; 17 Department of Internal Medicine, Ospedale per gli Infermi of Faenza, 48018 Faenza, Italy; fg.foschi@ausl.ra.it; 18 Infectious Diseases and Hepatology Unit, Azienda Ospedaliero-Universitaria of Parma, 43126 Parma, Italy; ebiasini@ao.pr.it; 19 Gastroenterology Unit, Ospedale Sacro Cuore Don Calabria, 37024 Negrar, Italy; alberto.masotto@sacrocuore.it; 20 Department of Clinical Medicine and Surgery, Hepato-Gastroenterology Unit, University of Napoli “Federico II”, 37024 Napoli, Italy; nardone@unina.it; 21 Department of Clinical and Experimental Medicine, Division of Medicine and Hepatology, University of Messina, 98122 Messina, Italy; raimondo@unime.it; 22 Department of Surgical and Medical Sciences, Gastroenterology Unit, Alma Mater Studiorum—University of Bologna, 40126 Bologna, Italy; francesco.azzaroli@unibo.it; 23 Department of Medical, Surgical and Experimental Sciences, Clinica Medica Unit, University of Sassari, Azienda Ospedaliero-Universitaria of Sassari, 07100 Sassari, Italy; gianpaolovidili@uniss.it; 24 Department of Clinical and Experimental Medicine, Hepatology and Liver Physiopathology Laboratory and Internal Medicine, University of Pisa, 56126 Pisa, Italy; maurizia.brunetto@unipi.it; 25 IRCCS Azienda Ospedaliero-Universitaria di Bologna, 40138 Bologna, Italy; franco.trevisani@unibo.it

**Keywords:** HCC, large, phenotypes, PVT, multifocality, albumin

## Abstract

**Simple Summary:**

Factors influencing the survival of hepatocellular carcinoma (HCC) patients include portal vein thrombosis (PVT), tumor numbers (multifocality), blood alpha-fetoprotein (AFP) levels, and the degree of liver damage (levels of blood bilirubin and albumin). However, the role of tumor size can be ambiguous. We therefore examined multiple clinical characteristics for their relationship with patient death and combined the three parameters with the greatest impact to create a tool to examine the characteristics and survival of patients with normal and abnormal levels of this three-parameter tool. In patients with large tumors, we found that normal levels of these three parameters—no PVT or multifocality plus normal blood albumin levels—were associated with longer survival than any group containing patients with PVT. This good-survival group could also be divided into two subgroups, differing in survival, based on blood AFP levels. This three-parameter tool might be prognostically useful in stratifying patients and management decisions.

**Abstract:**

Background. Hepatocellular carcinoma (HCC) factors, especially maximum tumor diameter (MTD), tumor multifocality, portal vein thrombosis (PVT), and serum alpha-fetoprotein (AFP), influence survival. Aim. To examine patterns of tumor factors in large HCC patients. Methods. A database of large HCC patients was examined. Results. A multiple Cox proportional hazard model on death identified low serum albumin levels and the presence of PVT and multifocality, with each having a hazard ratio ≥2.0. All combinations of these three parameters were examined in relation to survival. Using univariate Cox analysis, the combination of albumin >3.5 g/dL and the absence of both PVT and multifocality had the best survival rate, while all combinations that included the presence of PVT had poor survival and hazard ratios. We identified four clinical phenotypes, each with a distinct median survival: patients with or without PVT or multifocality plus serum albumin ≥3.5 (g/dL), with each subgroup displaying high (≥100 IU/mL) or low (<100 IU/mL) blood AFP levels. Across a range of MTDs, we identified only two significant trends, blood AFP and platelets. Conclusions. Patients with large HCCs have distinct phenotypes and survival, as identified by the combination of PVT, multifocality, and blood albumin levels.

## 1. Introduction

An increase in hepatocellular carcinoma (HCC) size is typically regarded as a poor prognostic factor, even though some large size HCCs can have an excellent prognosis [1,2,3]. However, many HCCs manifest an increase in tumor aggressiveness factors as size or maximum tumor diameter (MTD) increases [4,5]. It has recently been considered that MTD may not be that important when considered as an isolated parameter, as an increase in post-surgical treatment recurrences may be due to factors other than tumor size alone [2,6]. These differing findings suggest that there may be several phenotypes within the group of patients with large HCCs. In this paper, this idea was investigated by selecting three parameters that have high hazard ratios (HR) on death and using this three-parameter model to identify clinical patterns of HCC with better or worse prognosis, particularly by focusing only on HCCs with large MTD, in order to minimize any effect of tumor size. We found that the presence of macroscopic portal vein invasion (PVT) results in the worst prognosis, but in the absence of PVT, the presence or absence of elevated serum alpha-fetoprotein (AFP) levels was associated with two different phenotypes, each with different clinical features and prognosis.

## 2. Results

### 2.1. Development of a Three-Parameter Prognostic Group

A multiple Cox proportional hazard model for death was calculated for our HCC patients on common liver function tests, blood count, and HCC parameters (PVT, tumor multifocality, and serum AFP levels). We found that blood albumin levels and the presence of portal vein thrombosis (PVT) and tumor multifocality all had hazard ratios ≥2.0 (Table 1).

These three parameters were then combined in all eight possible combinations, and each combination was examined in relation to patient survival time using both Kaplan–Meier analysis and univariate Cox regression analysis (Table 2).

The results show that the longest survival (median 26 months) was for the combination of absent PVT and absent tumor multifocality plus normal blood albumin levels (>3.5 g/dL), and this was used as the reference univariate hazard ratio. Patients who also had absence of PVT and either no multifocality plus low (abnormal) albumin levels (median survival 15 months) or who had multifocality plus high (normal) albumin levels (median survival 15 months) had survival values that were significantly lower than the reference value and had HRs (hazard ratios) of 1.91 and 1.42, respectively, which were significantly different from the reference. All groupings of the three-parameter group that included the presence of PVT had low survival and HR values.

### 2.2. Clinical Correlates of the Three-Parameter Groups

The three-parameter combinations of the presence of PVT and multifocality plus low serum albumin (poor prognosis) versus the absence of PVT and absence of multifocality plus high serum albumin (good prognosis) were then compared for their correlative clinical characteristics and patient survival, each group being dichotomized according to low serum AFP (<100 IU/mL) or high serum AFP (≥100 IU/mL) levels (Table 3).

Results were similar across a range of MTDs, but we chose to present only the narrow range of 8–11 cm or large MTD patients with the aim of excluding any effect of tumor size/MTD itself, which has been previously shown to influence HCC biology [5]. Table 3 shows the clinical characteristics and survival of four groups of patients (two groups of three-parameter patients, each dichotomized according to high or low AFP values). The left two columns show a comparison of the two three-parameter groups, all in patients with low serum AFP levels. Levels of AFP, hemoglobin, aspartate aminotransaminase (AST), alkaline phosphatase (ALKP), total bilirubin, and high-density lipoprotein (HDL) cholesterol levels were significantly different between the two low-AFP groups, as well as survival at 1, 2 and 3 years (bottom 3 rows). However, a different result was found when the two groups were compared for patients who all had high serum AFP (≥100 IU/mL) levels. For patients with high levels of AFP, in addition to MTD, only total bilirubin, hemoglobin, and AST levels were significantly different between these two subsets, and although there were significant survival differences at 1 and 2 years (only), the survival rates were much lower than the low-AFP groups. Significance levels (*p* values) are shown on the right-hand two columns for comparisons of high-versus-low AFP subsets within the good-survival three-parameter groups (column (a) vs. (c)) and within the poor-survival three-parameter groups (column (b) vs. (d)). For the high vs. low AFP comparisons within the good-survival groups (column (a) vs. (c)), significant differences were found for serum AFP levels, ALKP and AST, as well as 1-year survival. For the high vs. low AFP comparisons within the poor-survival groups (column (b) vs. (d)), significant differences were only found for serum AFP levels and white blood counts and not for survival time. Thus, different AFP levels were associated with differences in the good-survival three-parameter groups but with only a few parameters in the poor-survival three-parameter groups.

### 2.3. Clinical Patterns for Three-Parameter Groups Associaedg with Different MTD Bands

We then evaluated the clinical patterns of patients with different ranges of tumor sizes (MTD) for each three-parameter group, using both the Kruskal–Wallis rank test and the test for trend. For the good-survival three-parameter group (Table 4A), blood parameter levels that were significantly different with increases in MTD were blood platelet counts, as previously found [7,8], and levels of AFP, ALT, AST, low-density lipoproteins (LDL) cholesterol, and total cholesterol. For the poor-survival three-parameter group (Table 4B), blood parameter levels that were significantly different as MTD increased were only blood platelet counts and AST (and total cholesterol levels for trend).

**Figure 1:**
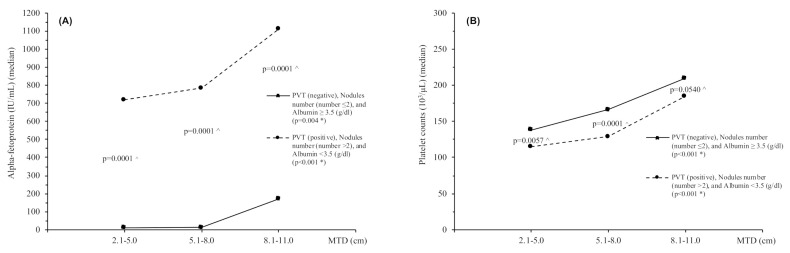
Trends of median values for serum AFP levels (**A**) and platelet counts (**B**) in relation to MTD categories for patients with PVT (negative), nodule number (≤2) and albumin ≥3.5 (g/dL) vs. patients with PVT (positive), nodule number (>2) and albumin <3.5 (g/dL), respectively. * Test for trend, ^ Kruskal–Wallis rank test. Abbreviations: MTD, maximum tumor diameter; AFP, alpha-fetoprotein; Plt, Platelet counts.

Survival decreased significantly as MTD increased in the good-survival three-parameter group but not in the poor-survival three-parameter group (Table 4A,B, lower part of each table). Figure 1 shows the changes in trends and levels of blood AFP (A) and blood platelets (B) with increasing MTD in the two three-parameter groups. The trends in the changing AFP and platelet levels are significant, as are their differences between each of the three-parameter groups.

## 3. Discussion

Many factors influence survival in HCC patients, including size, the presence of PVT, degree of tumor differentiation, level of serum AFP, tumor responses to therapy [9], and the presence of specific anatomical (and thus molecular) subtypes of HCC [10,11,12]. We have identified three parameters by their hazard ratios in a multiple Cox proportional hazard model on death and used them as a three-parameter group combination (PVT, tumor multifocality, and serum albumin levels, as shown in Table 1 and Table 2) to compare the differing patterns of clinical characteristics and survival (Table 3 and Table 4). To minimize any influence of tumor size itself, we used a narrow band of large MTD patients (8–11 cm). The three-parameter groups of these patients with large tumors differed significantly in their clinical characteristics and survival, but mainly for patients with low serum AFP levels. When the subsets of patients in the good-survival three-parameter group (absence of both PVT and multifocality plus normal/high serum albumin levels) were compared in terms of their AFP levels, as shown Table 3, column (a) vs. (c), only serum levels of ALKP and AST were significantly different. In the high-AFP patients, the three-parameter groups also had significant survival differences, but at much lower levels, as seen in Table 3, column (b) vs. (d). The lowest survival rates were found in patients with both PVT and multifocality plus low serum albumin levels, regardless of AFP values. We could thus identify four phenotypic patterns: patients with the presence of PVT and multifocality plus low serum albumin levels (poor survivors)—with either high or low AFP levels, and the opposite; namely, the absence of both PVT and multifocality plus normal serum albumin levels (good survivors)—with either high or low AFP levels. In practice, based on survival, probably only three patient groups are usefully identifiable, namely, good-survival patients without PVT or multifocality and with normal serum albumin levels, with either (a) low or (b) high AFP levels, and a third group (c), poor-survival patients with PVT and multifocality plus low serum albumin levels, regardless of serum AFP levels.

For comparison of patients with different MTD groups, patients were arranged according to 3 cm band groupings of 2.1–5.0, 5.1–8.0, and 8.1–11 (Table 4). The good-survival three-parameter groups nevertheless had significantly decreased survival as MTD increased, together with correspondingly significant increases in both blood AFP and platelet values. However, the poor-survival three-parameter group did not show survival differences for differing MTDs, probably because the survival values for all patients were so poor. Despite this, both good- and poor-survival three-parameter groups showed significant trends for both blood AFP and platelet counts as MTD increased (Figure 1). Thus, both AFP and platelet levels were associated with HCC growth, as shown elsewhere [5,7,8]. We considered the possibility that there might be selection bias, with “good” prognosis patients being selectively treated with surgically curative approaches. However, only a minority of patients were eligible for surgery, and the large size excluded almost all of them from transplant. Therefore, we think there was minimal selection bias in the analysis.

These findings are actually incorporated in and straddle the Barcelona clinic liver cancer (BCLC) classification system, which has stage A (single tumor of any size) as our good-prognosis group (absence of multifocality or PVT), stage B (multifocality) as part of our poor-prognosis group, and stage C (presence of PVT) as part of our poor-prognosis group. Thus, we applied the BCLC stages B and C together to large tumors (multifocality or PVT), and we have instead shown that the good-prognosis group of large MTD patients (BCLC stage A) can be refined into two prognostically separate and identifiable subgroups based on levels of serum AFP. AFP levels are not part of BCLC, but they an important part of decision making regarding HCC patient selection for liver transplants.

There are limitations to this study. As HCC patient criteria for liver transplant are being slowly extended, it would be advisable in future to include large tumor size resected [2] or transplanted [6] patients in survival analyses. Furthermore, the relationship between MTD as a key variable and other tumor parameters, such as PVT or multifocality, was not explored here, although others have begun to do so and suggest that MTD is not particularly important for prognosis on its own, but rather that large MTD may be a surrogate for other, negative prognostic factors [4,5,6].

The mechanisms underlying the roles of either blood AFP or platelet levels in HCC growth are not entirely clear, but they have been investigated to some extent. Thus AFP, which was first discovered 55 years ago by Tatarinov experimentally and then by Abelev clinically [13], has retained its place in HCC diagnosis and as a marker of response to therapy and for prognosis [14]. However, its mechanism of action in HCC growth and aggressiveness is far from clear. Recent studies have suggested that it has actions on growth signaling pathways, transcription, apoptosis control, immune modulation, and even in both metastasis development and direct HCC growth stimulation [15,16,17,18,19,20,21,22,23,24,25,26,27,28,29]. Platelets have been shown to be associated with HCC growth due to the synthesis and release of tumor growth factors, including PDGF, FGF, VEGF, and immune cytokines that play a role in immune modulation [30,31,32]. Platelets are thus also viewed as key factors in the tumor microenvironment [33].

Interestingly, tumor size has been recently de-emphasized as a key prognostic factor for liver transplant for HCC, it being claimed that the size-associated factors such as tumor differentiation, tumor multifocality, and PVT may be possibly more important than size itself [4,6], and this is also true for liver resection for HCC [2]. Nevertheless, more than one large HCC phenotype has been previously observed, often in association with higher blood platelet numbers and less severe cirrhosis [3,34,35]. Regardless, tumor size is still regarded as a key variable in HCC prognosis [36] and was seen to be significant for survival in Table 4, but only in the good-survival three-parameter group. Other parameters are also regarded as important for HCC survival, including serum levels of AFP [7,12,13,14,23] albumin, and bilirubin [37]. For patients with PVT, the level of MTD made no difference to survival.

## 4. Materials and Methods

### 4.1. Clinical Data Collection

We analyzed prospectively collected data in the Italian Liver Cancer (ITA.LI.CA) study group database of 2297 HCC patients collected at our multiple collaborating Italian centers with baseline tumor parameter data, including CT scan information on maximum tumor diameter (MTD), number of tumor nodules, presence or absence of PVT and serum AFP levels; complete blood counts; routine blood liver function tests, (total bilirubin, GGTP, ALKP, albumin, AST, and ALT); and demographics and survival information, as previously reported [7]. The ITA.LI.CA database management conforms to Italian legislation on privacy, and this study conforms to the ethical guidelines of the Declaration of Helsinki. Approval for this retrospective study on de-identified HCC patients was obtained from the Institutional Review Board of the participating centers. The study design was approved (protocol n. 99/2012/O/Oss) by the Independent Ethic Committee of S. Orsola-Malpighi hospital of Bologna, which operates as coordinating center of the ITA.LI.CA network. In all the participating centers, data inclusion in the ITA.LI.CA registry was approved by the local ethics committees.

### 4.2. Statistical Analysis

Patient characteristics are reported as mean ± standard deviation (M ± SD) or as median for continuous variables, and as frequencies and percentages (%) for categorical variables.

Normal distributions of quantitative variables were tested using the Kolmogorov–Smirnov test.

For testing the associations between groups, the chi-square test for categorical variables was used; when the variables were not normally distributed, the Wilcoxon rank sum test and Kruskal–Wallis rank test were used for continuous variables when necessary.

The test for trend was performed to evaluate the trend between the examined categorical levels for a continuous variable as a clinical and tumor parameter.

The proportion test was applied to evaluate the statistical differences between the parameters as category for each level of ordered maximum tumor diameters (MTDs, cm).

To evaluate the variation in the increase in the median of AFP or the percentage of PVT (+) and multifocality, the equation of the interpolating line for each modification of variation of the increase was used.

The variation in the medians of AFP, percentage of PVT (+), and percentage of multifocality (*n* > 2) in relation to the increase in MTD was calculated both as percentage variation from a previous value of parameters analyzed as increase in tumor size compared to the reference band. This proportionality factor represents how many times the value of the single factor increased with increasing MTD when compared to the first category of MTD.

To examine the time between initiation of study and a subsequent event, the non-parametric Kaplan–Meier method was used to explore survival probability, and the log-rank test was applied to evaluate the equality of survival among categories.

The Cox model was used because it is a statistical technique employed to explore the relationship between the survival of a patient and singular or several explanatory variables, and because it also allows for the estimation of the hazard risk (HR) of survival for an individual, given their prognostic variables (measured as continuous or categorical).

The Cox proportional hazard model was fitted to the data, and the proportional hazard assumption was evaluated by means of Schoenfeld residuals (SRT).

All models for fitting were evaluated by means of Akaike Information Criteria (AIC) and Bayesian information criterion (BIC).

Risk estimators were expressed as hazard ratios (HRs) and 95% confidence interval (95% CI). In the models, the multicollinearity was evaluated through the variance inflation factor (VIF), using a score of 2 as cut-off for exclusion.

When testing the null hypothesis of no association, the probability level of error at two tails was 0.05. All of the statistical computations were made using STATA, StataCorp. 2019. Stata Statistical Software: Release 16. College Station, TX: StataCorp LLC.

## 5. Conclusions

A combination of three parameters in routine clinical practice, including the presence or absence of PVT and tumor multifocality plus blood albumin levels, was used as a tool that identified distinct subsets of patients with large-sized HCC with typical clinical characteristics and survival. Patients with large HCCs are thus heterogenous, and some have prolonged survival.

## Figures and Tables

**Table 1:** Multiple Cox proportional hazard models for death on clinical parameter categories in hepatocellular carcinoma (HCC) patients.

	HR *	se(HR)	*p*	95% CI
Platelets (10^3^/μL)				
<100 (Ref. category)	1	--	--	--
≥100	0.996	0.050	0.94	0.903 to 1.099
Albumin (g/dL)				
≥3.5 (Ref. category)	1	--	--	--
<3.5	2.00	0.10	<0.001	1.81 to 2.21
Bilirubin (mg/dL)				
<1.2 (Ref. category)	1	--	--	--
≥1.2	1.54	0.07	<0.001	1.41 to 1.68
GGT (IU/mL)				
<100 (Ref. category)	1	--	--	--
≥100	1.24	0.08	0.001	1.09 to 1.40
AFP (IU/mL)				
<100 (Ref. category)	1	--	--	--
≥100	1.91	0.09	<0.001	1.74 to 2.09
ALKP (IU/L)				
<100 (Ref. category)	1	--	--	--
≥100	1.17	0.09	0.04	1.00 to 1.37
AST (IU/L)				
≤40 (Ref. category)	1	--	--	--
>40	1.60	0.09	<0.001	1.44 to 1.78
Hemoglobin (g/dL)				
≥13 (Ref. category)	1	--	--	--
<13	1.39	0.06	<0.001	1.27 to 1.52
PVT				
Negative (Ref. category)	1	--	--	--
Positive	2.03	0.10	<0.001	1.84 to 2.24
Nodule number (number)				
(n ≤ 2) (Ref. category)	1	--	--	--
(n > 2)	2.03	0.09	<0.001	1.85 to 2.23

* HR, hazard ratio. Abbreviations: MTD, maximum tumor diameter; GGTP, gamma glutamyl transpeptidase; AFP, alpha-fetoprotein; ALKP, alkaline phosphatase; AST, aspartate aminotransaminase; PVT, portal vein thrombosis.

**Table 2:** Kaplan–Meier analysis and Cox regression for PVT and multifocality and albumin combinations in HCC patients.

Parameters				Kaplan-Meier Analysis		Univariate Cox Regression	
PVT	Nodule Number (Number)	Albumin (g/dL)	*n*	Survival Time (Median) Mean ± SE	Log-Rank *p*-Value	Univariate HR (95% CI)	HR *p*-Value
Negative	(*n* ≤ 2)	≥3.5	362	(26) 37.91 ± 35.96	(Ref. category)	(Ref. category) ^#^	--
Negative	(*n* ≤ 2)	<3.5	296	(15) 23.96 ± 27.68	<0.0001	1.91 (1.56 to 2.34)	<0.001
Negative	(*n* > 2)	≥3.5	60	(15) 23.28 ± 22.79	<0.0001	1.42 (1.20 to 1.69)	<0.001
Negative	(*n* > 2)	<3.5	103	(5) 10.92 ± 15.79	<0.0001	1.43 (1.58 to 1.89)	<0.001
Positive	(*n* ≤ 2)	≥3.5	147	(10) 19.15 ± 22.11	<0.0001	1.25 (1.17 to 1.33)	<0.001
Positive	(*n* ≤ 2)	<3.5	232	(6) 12.07 ± 17.17	<0.0001	1.33 (1.28 to 1.39)	<0.001
Positive	(*n* > 2)	≥3.5	88	(7) 13.06 ± 16.22	<0.0001	1.27 (1.21 to 1.33)	<0.001
Positive	(*n* > 2)	<3.5	166	(5) 9.05 ± 15.69	<0.0001	1.30 (1.26 to 1.35)	<0.001

^#^ Reference category for each level of combined parameters. Abbreviations: MTD, maximum tumor diameter; PVT, portal vein thrombosis; BCLC, Barcelona clinic liver cancer.

**Table 3:** Clinical and tumor parameter values according to combinations of PVT, multifocality, and albumin, subdivided into AFP in categories in HCC patients with MTD (8–11) cm.

	AFP < 100 IU/mL			AFP ≥ 100 IU/mL			Comparisons	
Parameters ^§^	PVT (Negative) and # Nodules (≤2) and Albumin ≥3.5 (g/dL)	PVT (Positive) and # Nodules (>2) and Albumin <3.5 (g/dL)	*p* *	PVT (Negative) and # Nodules (≤2) and Albumin ≥3.5 (g/dL)	PVT (Positive) and # Nodules (>2) and Albumin <3.5 (g/dL)	*p* *	*p* *	*p* *
	*n* = 66	*n* = 28		*n* = 61	*n* = 57			
	(a)	(b)		(c)	(d)		(a) vs. (c)	(b) vs. (d)
Gender (Male) (%)	57 (86.36)	24 (85.71)	0.93 ^^^	52 (85.25)	46 (80.70)	0.51 ^^^	0.86 ^^^	0.57 ^^^
Age (y) (M ± SD) #	64.67 ± 12.25	64.57 ± 13.69	0.98	61.24 ± 13.85	57.96 ± 13.51	0.14	0.20	0.04
MTD (cm)	9.00	8.4	0.24	9.00	9.5	0.006	0.75	0.002
AFP (IU/mL)	5.88	17.93	0.001	1524.00	3769.32	0.17	<0.0001	<0.0001
Total Bilirubin (mg/dL)	0.85	1.10	0.02	0.95	1.70	0.0001	0.25	0.37
Platelet counts (10^3^/μL)	212.00	204.00	0.50	190.00	157.00	0.11	0.22	0.21
ALKP (IU/L)	220.00	385.00	0.02	287.00	227.00	0.21	0.003	0.18
GGTP (IU/L)	131.50	240.00	0.07	200.00	265.50	0.08	0.12	0.52
White Blood Cells	6500.00	6010.00	0.61	4260.00	3170.00	0.15	0.18	0.04
Hemoglobin (g/dL)	13.30	10.90	<0.0001	14.00	11.80	<0.0001	0.09	0.31
ALT (IU/L)	40.00	54.50	0.44	42.00	54.00	0.43	0.43	0.57
AST (IU/L)	43.50	69.00	0.0009	56.00	112.00	0.002	0.009	0.25
HDL (mg/dL)	49.00	23.00	0.001	39.00	37.00	0.20	0.13	0.28
LDL (mg/dL)	100.00	85.00	0.63	100.00	77.00	0.30	0.68	0.46
Total Cholesterol (mg/dL)	159.00	143.00	0.75	156.00	131.00	0.24	0.52	0.21
Survival Probability at time (%)								
1 y	73.53	33.33	0.01 ^^^	46.43	8.70	0.003 ^^^	0.03	0.07 ^^^
2 y	50.00	8.33	0.01 ^^^	28.57	4.35	0.02 ^^^	0.09	0.63 ^^^
3 y	44.12	0.00	0.005 ^^^	14.29	4.35	0.23 ^^^	0.01	0.46 ^^^

^§^ All values as median; # value as mean and standard deviation; * Wilcoxon rank-sum (Mann–Whitney) test; ^^^ chi-square test. Abbreviations: PVT, portal vein thrombosis; MTD, maximum tumor diameter; AFP, alpha-fetoprotein; ALKP, alkaline phosphatase; GGTP, gamma glutamyltranspeptidase; ALT, alanine transaminase; AST, aspartate aminotransaminase; HDL, high-density lipoprotein; LDL, low-density lipoproteins.

**Table 4:** Clinical and tumor parameter values subdivided by MTD categories in patients with PVT (negative), nodule number (number ≤2), and albumin ≥3.5 (g/dL) (**A**), and with PVT (positive), nodule number (number >2), and albumin <3.5 (g/dL) (**B**).

Parameter	MTD (cm)				
	(2.1–5.0)	(5.1–8.0)	(8.1–11.0)	*p* *	*p* ^§^
	(*n* = 1787)	(*n* = 395)	(*n* = 115)		
(**A**)					
AFP (IU/mL) median	10.90	13.10	171.00	0.0001	<0.001
Total Bilirubin (mg/dL) median	0.90	0.80	0.90	0.07	0.14
Platelet counts (10^3^/μL) median	138.00	166.00	210.00	0.0001	<0.001
ALKP (IU/L) median	220.00	220.00	220.00	0.33	0.98
GGTP (IU/L) median	100.00	100.00	140.50	0.48	0.39
White Blood Cells median	4670.00	4940.00	5670.00	0.58	0.31
Hemoglobin (g/dL) median	13.60	13.70	13.70	0.58	0.29
ALT (IU/L) median	42.00	40.00	44.00	0.01	0.09
AST (IU/L) median	44.00	41.00	56.00	0.002	0.15
HDL (mg/dL) median	43.00	45.00	43.70	0.60	0.42
LDL (mg/dL) median	85.00	100.00	111.00	0.004	0.001
Total Cholesterol (mg/dL) median	151.00	167.00	168.00	0.002	0.001
Survival Probability at time (%)					
1 y	847 (86.16)	164 (79.61)	30 (57.69)	<0.001 ^^^	<0.0001
2 y	615 (62.56)	113 (54.85)	21 (40.38)	0.001 ^^^	0.0003
3 y	447 (45.47)	77 (37.38)	15 (28.85)	0.01 ^^^	0.002
	MTD (cm)				
	(2.1–5.0)	(5.1–8.0)	(8.1–11.0)	*p* *	*p* ^§^
	(*n* = 77)	(*n* = 125)	(*n* = 272)		
(**B**)					
AFP (IU/mL) median	720.00	785.79	1111.14	0.12	0.04
Total Bilirubin (mg/dL) median	2.00	2.00	2.10	0.77	0.48
Platelet counts (10^3^/μL) median	115.00	129.00	185.00	0.0001	<0.001
ALKP (U/L) median	220.00	220.00	309.50	0.17	0.09
GGTP (U/L) median	200.00	199.00	206.00	0.59	0.39
White Blood Cells median	3810.00	3500.00	3305.00	0.87	0.89
Hemoglobin (g/dL) median	11.80	11.70	11.40	0.76	0.76
ALT (U/L) median	60.00	60.00	64.00	0.85	0.66
AST (U/L) median	92.50	110.00	129.50	0.01	0.005
HDL (mg/dL) median	34.00	24.50	26.00	0.37	0.25
LDL (mg/dL) median	100.10	81.50	112.50	0.15	0.11
Total Cholesterol (mg/dL) median	130.50	131.00	158.00	0.14	0.05
Survival Probability at time (%)					
1 y	7 (17.50)	10 (18.18)	9 (16.98)	0.99 ^^^	0.94
2 y	2 (5.00)	5 (9.09)	3 (5.66)	0.68 ^^^	0.96
3 y	1 (2.50)	2 (3.64)	2 (3.77)	0.94 ^^^	0.75

Abbreviations: MTD, maximum tumor diameter; PVT, portal vein thrombosis; AFP, alpha-fetoprotein; ALKP, alkaline phosphatase; GGTP, gamma glutamyltranspeptidase; AST, aspartate aminotransaminase; ALT, alanine transaminase; HDL, high-density Lipoprotein; LDL, low-density Lipoproteins. * Kruskal–Wallis rank test; ^^^ chi-square test; ^§^ test for trend.

## Data Availability

Data available on request due to restrictions of patient privacy.
